# Supplementary material for: Soil Bacterial Diversity and Potential Functions Are Regulated by Long-Term Conservation Tillage and Straw Mulching
Source: Microorganisms. 2020 Jun 2;8(6):836. doi: 10.3390/microorganisms8060836 (PMC7355473; doi:10.3390/microorganisms8060836)
Supplement: Supplementary file 1 [file microorganisms-08-00836-s001.pdf]

Supplementary Material

Soil Bacterial Diversity and Potential Functions Are Regulated by Long-Term Conservation Tillage and Straw Mulching

Chang Liu [1, 2], Lingling Li [1,2,*], Junhong Xie [1,2], Jeffrey A. Coulter [3], Renzhi Zhang [1,4], Zhuzhu Luo [1,4], Liqun Cai [1,4], Linlin Wang [1,2], Subramaniam Gopalakrishnan [5]

[1] Gansu Provincial Key Laboratory of Aridland Crop Science, Gansu Agricultural University, Lanzhou 730070, China; liuc@gsau.edu.cn (C.L.); lill@gsau.edu.cn (L.L.) xiejh@gsau.edu.cn (J.X.); zhangrz@gsau.edu.cn (R.Z.); luozz@gsau.edu.cn (Z.L.); wangll@gsau.edu.cn (L.W.)
[2] College of Agronomy, Gansu Agricultural University, Lanzhou 730070, China;
[3] Department of Agronomy and Plant Genetics, University of Minnesota, St. Paul, MN 55108, USA; jeffcoulter@umn.edu (J.C.)
[4] College of Resource and Environment, Gansu Agricultural University, Lanzhou 730070, China;
[5] International Crops Research Institute for the Semi-Arid Tropics (ICRISAT), Patancheru, Hyderabad, Telangana 502324, India; s.gopalakrishnan@cgiar.org (S.G.)
* Correspondence: lill@gsau.edu.cn (L.L.)

Table S1. Detailed sequencing depth results of soil samples under different tillage treatments.

Sample[a]	Clean_tags	Valid_tags	OTU_counts	Goods_coverage
NT.1	39841	32461	2905	0.976
NT.2	41562	33206	2843	0.975
NT.3	40998	33025	2926	0.975
NTS.1	40646	32499	2893	0.977
NTS.2	41790	35385	2385	0.977
NTS.3	41848	36654	2433	0.973
T.1	40409	33915	1909	0.979
T.2	40354	33481	1952	0.979
T.3	38813	32782	2426	0.976
TS.1	39001	31547	2880	0.977
TS.2	38776	31342	2763	0.975
TS.3	41335	34527	2649	0.975

[a] NT, no-tillage with all crop residue removed at harvest; NTS, no-tillage with crop residue chopped and spread evenly on the soil surface; T, conventional tillage with all crop residue removed at harvest; TS, conventional tillage with all crop residue chopped, spread evenly on the soil surface, and incorporated into the soil via plowing.

Table S2. Relative abundance of soil bacterial taxonomic composition at phylum level for all samples as affected by tillage and crop residue management treatments.

Taxonomy (%)	Sample[a]											
	NT.1	NT.2	NT.3	NTS.1	NTS.2	NTS.3	T.1	T.2	T.3	TS.1	TS.2	TS.3
Proteobacteria	30.95	42.33	38.66	42.34	62.37	63.75	19.35	17.97	59.31	27.39	37.36	50.75
Bacteroidetes	25.90	11.70	16.23	17.71	9.06	10.37	41.34	49.27	8.27	29.55	25.64	15.83
Actinobacteria	25.92	27.95	28.27	22.15	17.65	11.47	17.61	19.59	19.57	20.57	25.71	21.50
Firmicutes	7.98	4.96	5.99	9.86	5.28	8.19	16.20	6.96	5.72	14.05	4.96	4.68
Gemmatimonadetes	5.90	8.25	6.63	4.48	2.96	3.60	3.69	4.13	4.77	4.87	3.67	4.27
Acidobacteria	1.64	2.60	2.27	1.82	1.21	1.23	1.04	1.09	1.23	1.46	1.27	1.17
Chloroflexi	0.27	0.32	0.40	0.32	0.25	0.12	0.21	0.23	0.26	0.35	0.32	0.24
Nitrospirae	0.23	0.39	0.31	0.22	0.18	0.15	0.19	0.16	0.23	0.21	0.15	0.14
Fusobacteria	0.14	0.09	0.12	0.20	0.37	0.35	0.00	0.25	0.07	0.22	0.09	0.16
JL_ETNP_Z39	0.14	0.29	0.13	0.18	0.08	0.08	0.06	0.08	0.04	0.08	0.04	0.07
Tenericutes	0.14	0.10	0.12	0.14	0.06	0.07	0.01	0.00	0.10	0.18	0.09	0.17
Cyanobacteria	0.18	0.26	0.15	0.08	0.08	0.02	0.03	0.04	0.06	0.03	0.03	0.06
Chlorobi	0.06	0.16	0.12	0.08	0.09	0.06	0.06	0.08	0.07	0.11	0.04	0.06
Fibrobacteres	0.12	0.12	0.11	0.06	0.06	0.14	0.08	0.05	0.05	0.04	0.08	0.07
Spirochaetae	0.00	0.02	0.02	0.03	0.10	0.23	0.00	0.00	0.00	0.04	0.08	0.39
Deferribacteres	0.06	0.02	0.08	0.11	0.03	0.02	0.00	0.00	0.02	0.16	0.07	0.04
Elusimicrobia	0.05	0.10	0.04	0.05	0.03	0.04	0.05	0.04	0.03	0.07	0.06	0.05
Verrucomicrobia	0.04	0.10	0.07	0.04	0.03	0.04	0.03	0.02	0.03	0.03	0.02	0.03
Saccharibacteria	0.01	0.00	0.01	0.01	0.03	0.01	0.00	0.00	0.02	0.01	0.04	0.01
Gracilibacteria	0.02	0.00	0.01	0.01	0.00	0.00	0.00	0.00	0.00	0.02	0.01	0.05
TM6	0.00	0.00	0.01	0.00	0.01	0.02	0.00	0.01	0.00	0.02	0.02	0.00
WCHB1_60	0.01	0.02	0.01	0.01	0.01	0.00	0.01	0.00	0.01	0.00	0.01	0.01
Planctomycetes	0.01	0.00	0.01	0.00	0.00	0.00	0.00	0.00	0.00	0.01	0.02	0.01
Parcubacteria	0.01	0.01	0.01	0.01	0.00	0.01	0.00	0.00	0.01	0.00	0.00	0.00
Armatimonadetes	0.00	0.01	0.01	0.00	0.00	0.00	0.00	0.00	0.00	0.00	0.00	0.00
SHA_109	0.00	0.01	0.00	0.00	0.00	0.00	0.00	0.00	0.00	0.01	0.00	0.00
Chlamydiae	0.00	0.00	0.00	0.00	0.00	0.00	0.00	0.00	0.00	0.01	0.00	0.00
Latescibacteria	0.00	0.00	0.00	0.00	0.01	0.00	0.00	0.00	0.00	0.00	0.00	0.00

Deinococcus_Thermus	0.00	0.00	0.00	0.00	0.00	0.00	0.00	0.00	0.00	0.00	0.00	0.00
Lentisphaerae	0.00	0.00	0.00	0.00	0.01	0.00	0.00	0.00	0.00	0.00	0.00	0.00
Caldiserica	0.00	0.00	0.00	0.00	0.00	0.00	0.00	0.00	0.00	0.00	0.00	0.00

[a] NT, no-tillage with all crop residue removed at harvest; NTS, no-tillage with crop residue chopped and spread evenly on the soil surface; T, conventional tillage with all crop residue removed at harvest; TS, conventional tillage with all crop residue chopped, spread evenly on the soil surface, and incorporated into the soil via plowing.

Table S3. Redundancy analysis results of soil physiochemical properties affecting the distribution of dominant phyla and clustering of soil samples.

Soil property[a]	Explains (%)	Contribution (%)	*F*	*P*
SOC	52.0	63.6	10.8	0.002
NT	13.7	16.8	3.6	0.056
NH_4-N	11.2	13.7	3.8	0.048
pH	3.0	3.0	0.8	0.500
NO_3-N	0.9	1.1	0.3	0.798
Moisture	0.9	1.0	0.2	0.840
TP	0.7	0.1	0.1	0.886

[a] SOC, soil organic carbon; TN, total nitrogen; TP, total phosphorus.

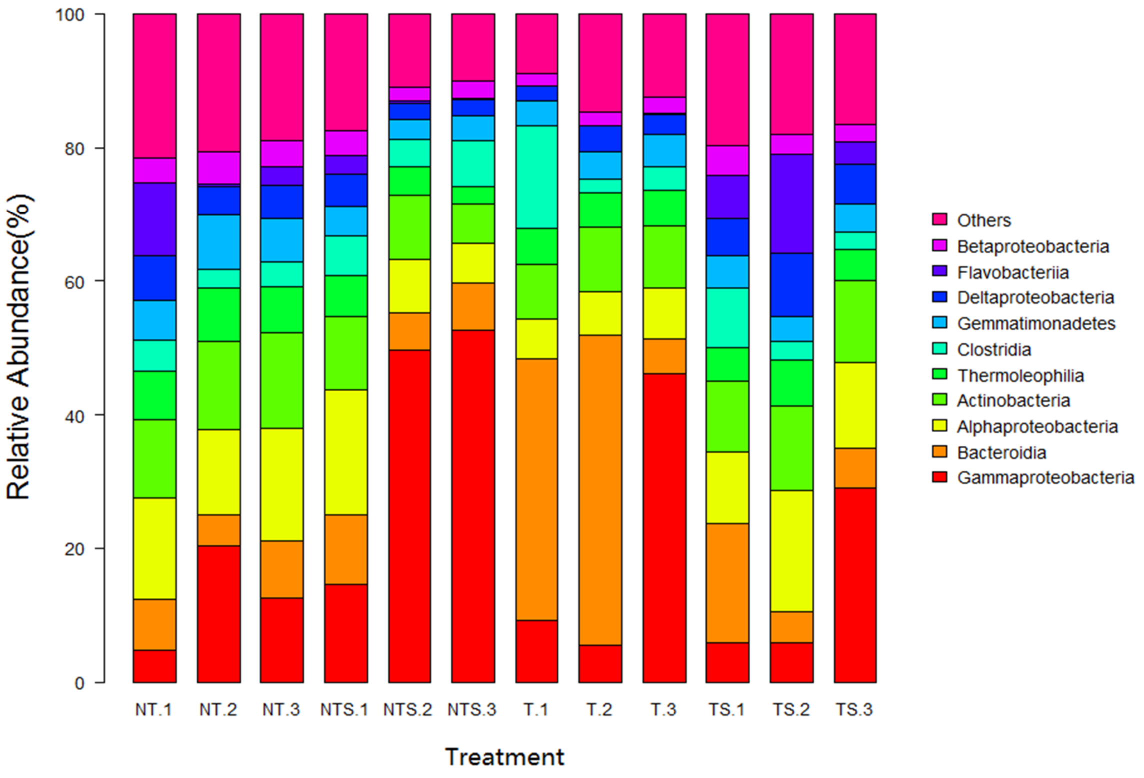

Figure S1. Relative abundance of top 10 soil bacterial class as affected by tillage and crop residue management treatments. NT, no-tillage with all crop residue removed at harvest; NTS, no-tillage with crop residue chopped and spread evenly on the soil surface; T, conventional tillage with all crop residue removed at harvest; TS, conventional tillage with all crop residue chopped, spread evenly on the soil surface, and incorporated into the soil during plowing. The numbers following the treatment name denote the sampling replications. For example, NT1, NT2, and NT3 means the soil sampling was taken from replicate 1, 2, and 3 of the field plots, respectively.
